# Time to bring patients to the core of care

**DOI:** 10.1016/j.lansea.2024.100459

**Published:** 2024-08-09

**Authors:** The Lancet Regional Health – Southeast Asia

In this issue of *The Lancet Regional Health – Southeast Asia*, Noronha and colleagues inform us about a patient advocacy group that works with patients with lung cancer to empower them with knowledge, resources, and support. Although not new, patient support groups have been working in India and the southeast Asia region to support and advocate for patients and their families, particularly those from marginalised groups, to reach available care in the best possible way. These groups, although important in bringing out the voices of patients, are not enough to make up for a system that is not currently oriented to provide care centred around patients.

People-centred care is an approach that stresses the involvement of patients, caregivers, and communities in the decision-making process, recognising their preferences, needs, and values. In India, the concept of people-centred care has been gaining attention, although its implementation is far from a reality. The National Health Policy 2017 emphasises the need for a people-centric approach. Accreditation bodies like the National Accreditation Board for Hospitals & Healthcare Providers include people-centred care principles in their standards. The PM-JAY scheme (national publicly funded health insurance scheme) emphasises people-centred care by reducing financial barriers. The National Digital Health Mission, another such initiative, aims to create a digital health ecosystem that supports integrated care. The state of Kerala in India, often praised for its advanced health-care system, has been implementing several such initiatives with people at its centre. Aardram Mission aims to transform the state's public health system to be more people-friendly and efficient, including the conversion of Primary Health Centres into Family Health Centres. eHealth Kerala is a digital health initiative that aims at facilitating better coordination of care and empowers patients by providing them access to their health information.

In India and several Asian countries, navigating complex and fragmented health systems can be challenging, particularly for those from rural areas, marginalised communities, or with limited financial resources. Health-care facilities are often concentrated in urban areas, leaving rural populations with limited access to quality care. Poor infrastructure, including inadequate transportation and poorly maintained roads, limited insurance coverage, and majority of out-of-pocket payments means that many patients cannot afford comprehensive care, especially for chronic and catastrophic illnesses. Low levels of health literacy also means that people cannot fully understand their health conditions, treatment options, and the importance of preventive care. Medical education in India has traditionally focused on clinical skills and biomedical knowledge, with less emphasis on people-centred aspects of care; involving patients in decision-making may also be perceived as a loss of authority or control over the treatment process.

Although patient advocacy groups, self-help groups, voluntary organisations, and NGOs have been working towards providing support to patients who find it difficult to navigate the health system and burdensome to continue care for catastrophic illness, they are merely filling a void left by a system that could not account for its patients. To reach the Sustainable Development Goals of equity and universal health coverage, we need a major shift in our approach towards care. According to WHO, we need to move from health systems designed around diseases and health institutions towards health systems designed for people. Continued efforts in education, resource allocation, and policy implementation are essential to fully align medical practice with the needs and preferences of patients. Implementing people-centred care in India is a complex challenge that requires addressing systemic, financial, educational, cultural, and technological barriers. India can only aspire to move closer to a health-care system responsive to the needs of its people, after addressing these challenges. The WHO framework for meaningful engagement of patients provides guidance on meaningful engagement and related approaches from a growing evidence base.

*The Lancet Regional Health – Southeast Asia* is committed to advocate for equity in access to health and care. We believe that a people-centred approach, combining scientific knowledge and lived experiences, is critical in achieving equity and universal health coverage goals. We invite researchers and practitioners to engage in research and care that is meaningful to the people to whom it matters the most.

